# Mass Spectrometry-Based Spatial Multiomics Revealed Bioaccumulation Preference and Region-Specific Responses of PFOS in Mice Cardiac Tissue

**DOI:** 10.1021/acs.est.4c09874

**Published:** 2025-01-22

**Authors:** Rui Shi, Yanyan Chen, Wenlong Wu, Xin Diao, Leijian Chen, Xingxing Liu, Haijiang Wu, Jianing Wang, Lin Zhu, Zongwei Cai

**Affiliations:** † State Key Laboratory of Environmental and Biological Analysis, Hong Kong Baptist University, Hong Kong SAR, 999077, China; ‡ Eastern Institute of Technology, Ningbo 315200, China

**Keywords:** PFOS, bioaccumulation, mass spectrometry imaging, spatial proteomics, Mass spectrometry analysis

## Abstract

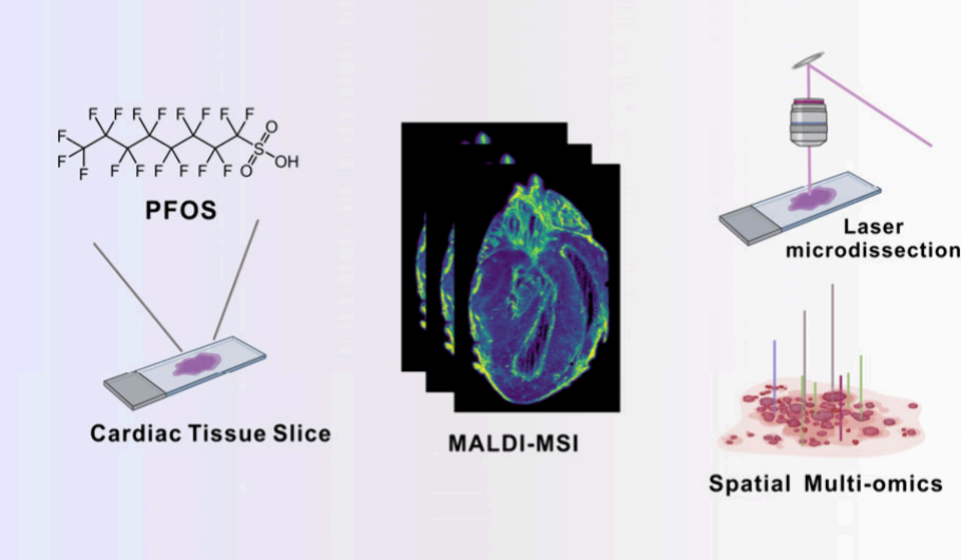

The distribution and bioaccumulation of environmental pollutants are essential to understanding their toxicological mechanism. However, achieving spatial resolution at the subtissue level is still challenging. Perfluorooctanesulfonate (PFOS) is a persistent environmental pollutant with widespread occurrence. The bioaccumulation behavior of PFOS is complicated by its dual affinity for phospholipids and protein albumin. It is intriguing to visualize the distribution preference of PFOS and investigate the differential microenvironment responses at a subtissue level. Herein, we developed a mass-spectrometry (MS)-based spatial multiomics workflow, integrating matrix-assisted laser desorption/ionization MS imaging, laser microdissection, and liquid chromatography MS analysis. This integrated workflow elucidates the spatial distribution of PFOS in mouse cardiac tissue, highlighting its preferential accumulation in the pericardium over the myocardium. This distribution pattern results in greater toxicity to the pericardium, significantly altering cardiolipin levels and disrupting energy metabolism and lipid transport pathways. Our integrated approach provides novel insights into the bioaccumulation behavior of PFOS and demonstrates significant potential for revealing complex molecular mechanisms underlying the health impacts of environmental pollutants.

## Abstract 2

Novel spatial multiomics workflow revealed that PFOS tends to accumulate and act more in the pericardium than in the myocardium.

## Introduction

Over 10,000 kinds of perfluoroalkyl and polyfluoroalkyl substances (PFASs) are used or generated as byproducts during various industrial production, leading to widespread environmental contamination and human exposure.^1−3^ As a representative legacy PFAS, perfluorooctanesulfonate (PFOS) has been known for its exceptional environmental persistence and strong bioaccumulation.^4−6^ Its exposure has been reported to cause various health issues including liver disease,^7^ chronic kidney disease,^8^ cancer,^9,10^ and cardiovascular conditions such as atherosclerosis, dyslipidemia, as well as increasing the risk of heart attack.^11−13^ Therefore, PFOS was the first PFAS to be added to Annex B of the Stockholm Convention on Persistent Organic Pollutants list, leading to global restrictions on its production and use.^14^

Like other PFASs, a strong affinity for serum albumin enables PFOS to distribute throughout the human body via blood circulation.^15^ Its affinity to phospholipids allows incorporation into cellular lipid bilayer and accumulation in lipid-rich tissues.^16−18^ The distribution of PFOS results from the combined effects of these properties. Extensive studies demonstrated that PFOS accumulates in the liver, kidneys, lungs, and hearts and even crosses the blood-brain barrier to accumulate in the brain, thereby affecting the function and homeostasis of these organs.^17^ However, research focusing on the subtissue distribution of PFOS is limited, due to technical difficulties and the complex interplay of its lipophilicity and protein-binding properties. Given that PFOS can interact with proteins involved in cholesterol metabolism and transport,^19^ it is interesting to investigate their spatial distribution and potential differential molecular effects in the cardiovascular system at a subtissue level.

To fill in this knowledge gap, we have developed a mass-spectrometry (MS)-based spatial multiomics workflow by integrating the MS-imaging (MSI), laser microdissection (LMD), and high-resolution MS-based multiomics analysis. Matrix-assisted laser desorption/ionization-mass spectrometry imaging (MALDI-MSI) is a powerful technology that enables unbiased, label-free molecular imaging of endogenous and exogenous substances directly on tissue sections.^20^ Its high sensitivity and specificity allow precise mapping of the spatial distribution of environmental pollutants and the corresponding metabolic disturbance caused.^21,22^ LMD is an advanced separation technique that allows for the contamination-free collection of the regions of interest from tissue sections using a focused laser beam. It emerges as a downstream complementary tool for various imaging techniques to isolate regions of interest for in-depth analysis.^23,24^ By coupling these two powerful techniques with liquid chromatography–mass spectrometry (LC/MS)-based proteomics and metabolomics analysis, it is possible to achieve comprehensive characterization of biological samples at multiple omics levels (proteomic, lipidomic, and metabolomic) with spatial resolution. This integrated workflow provides unprecedented insights into the complex interactions and dynamic adaptations of various tissue microenvironments with spatial distribution information, thereby allowing for a more accurate understanding of responses to external perturbations such as environmental pollutants. In this study, we employ the MS-based spatial multiomics workflow to characterize the spatial distribution of PFOS in mouse cardiac tissue and its impact on the proteome, metabolome, and lipidome levels. We revealed an unexpected accumulation pattern and region-specific toxic mechanisms of PFOS and demonstrated that this strategy has great potential in elucidating the molecular complexity of other pollutant subtypes.

## Materials and Methods

### Chemicals and Materials

Perfluorooctanesulfonate (PFOS, ≥99%), Dihydroxybenzoic acid (DHB, ≥98%), *N*-naphthylethylenediamine dihydrochloride (NEDC), Norharmane (Nor, ≥98%), sodium chloride (NaCl), and hematoxylin and eosin (H&E) were purchased from Sigma-Aldrich (St. Louis, MO). Chemical reagents unless specifically mentioned, including Methanol, isopropanol (IPA), chloroform, and acetonitrile (ACN), were HPLC-grade (Merck, Darmstadt, Germany). Milli-Q water was obtained from an ultrapure water purification system (Millipore, Billerica, MA, USA). Indium tin oxide-coated glass slides were purchased from Delta Technologies, Limited (Colorado, USA).

### Animal Experiment

The procedures of animal experiments and protocols were approved by the Committee on the Use of Human & Animal Subjects in Teaching and Research of Hong Kong Baptist University. Male ICR mice (4–5 weeks old) were obtained from the Laboratory Animal Service Centre at the Chinese University of Hong Kong. A total of 12 mice were used (6 per group for control and treatment). All mice were maintained on standard mouse chows, and sterile water and diets were provided ad libitum. Mice were housed under controlled conditions (22 ± 2 °C, 45 ± 10% relative humidity, and a 12-h light-dark cycle each day), and their body weight was monitored weekly.

The exposure experiment was conducted following our previous protocol.^25^ To observe the bioaccumulation pattern and initial toxicological response at heart tissue before causing significant organic pathology,^26^ a short time of 14 days exposure was applied. At the start of the experiment, mice were randomly assigned to either a control or a PFOS-treated group (*n* = 6 for each group). PFOS was dissolved in 2% Tween 80 and administered to mice via oral gavage once daily for 14 days at 2 mg/kg. Control mice received an equivalent volume of 2% Tween 80 without PFOS. The mice showed no significant differences in body weight throughout the exposure period, and no significant myocardial damage or evidence of inflammatory response was observed using hematoxylin and eosin (H&E) staining (Figure S1). After the exposure period, the mice were sacrificed by cervical dislocation after deep anesthesia by isoflurane.^25^ Heart samples were harvested without cardiac perfusion as we previously reported.^27^ The whole hearts were collected on an ice platform and instantly washed with ice-cold saline three times to remove residual blood. The samples were then snap-frozen in liquid nitrogen before being transferred to storage at −80 °C until further use.

### MALDI-MSI Analysis

The Mice hearts were sectioned at −20 °C on a cryo-Star Nx70 Cryostat (ThermoFisher Scientific, USA) at a 10 μm thickness and thaw mounted on indium tin oxide-coated glass slides. The slides were dried in a vacuum desiccator for 30 min before the matrix application. Nor and NEDC were prepared in MeOH at 5 and 10 mg/mL, respectively. Matrix was deposited directly on ITO slides by using the HTX H5 sprayer (HTX Technologies, Chapel Hill, NC, U.S.A.).^21^ The instrumental parameters employed were as follows: a flow rate of 0.05 mL/min; a velocity of 2000 mm/min; a tracking spacing of 2 mm; a pressure of 10 psi; a total of 12 spray cycles; and a drying time of 25 s. The temperature of the spray head was set to 66 °C.

MS images were acquired on a RapifleX MALDI Tissuetyper mass spectrometer (Bruker Daltonics, Germany) equipped with a Smartbeam 3D laser (355 nm) at a repetition rate of up to 10 kHz. Mass spectra were acquired at a mass range of *m*/*z* 80–2000 in negative ion reflector modes by averaging signal from 1000 shots, using a detector gain at 3.0 × 2810 V and 56% laser power. Key parameters were optimized and fixed throughout the experiment, including a reflector voltage of 20.84 kV, a lens voltage of 11.00 kV, an ion source voltage of 20 kV, and a pulsed ion extraction time of 100 ns. MALDI-Imaging Mass Spectrometry (IMS) was performed with a spatial resolution of 30 μm. Calibration was performed using external standards (Bruker Daltonics) as reported. MSI raw data were processed using SCiLS Lab MVS (version 2023b premium 3D) as previously described.^21,28^ Data were normalized by total ion count (TIC), and weak denoising was applied to all ion images. Lipids were assigned by searching Lipid Maps (https://www.lipidmaps.org) with a 10-ppm mass tolerance.

The bisecting k-means algorithm was employed to identify lipid metabolic differences between the two groups of cardiac tissue sections. SCiLS Lab (version 2023b premium 3D, Germany) was used for analysis using default settings as previously published.^26^ First, MSI raw data was imported into the software. Spectra were normalized using the total ion count (TIC) method, and weak denoising was applied to process all ion images. Automatic peak picking was performed by using the default segmentation pipeline. After peak picking and weak denoising, we selected the identified lipids as a cluster of peaks, and the lipid spectral information extracted from each point was thus clustered based on their spectral similarity.^29^ The spatial segmentation results are presented as spatial segmentation maps and corresponding hierarchical dendrograms composed of clusters, with pseudo colors assigned to pixels belonging to each cluster.

### Laser Microdissection

The adjacent heart sections (10 μm thickness) for the LMD were mounted onto polyethylene naphthalate (PEN)-membrane glass slides (Leica). Leica LMD 7 system (Leica Microsystems GmbH, Wetzlar, Germany) was used to collect tissue contours. Tissue was cut under a 20× objective in brightfield mode. The following laser settings were used for the 20× objective (HC PL FL L 20*x*/0.40 CORR): power 45, aperture 1, speed 8, middle pulse count 1, final pulse 1, head current 80%, pulse frequency 240, and offset 101. The pericardium and myocardium were dissected until the total dissected area reached 1.5 mm^2^ and collected into 0.5 mL protein low-binding tubes (Eppendorf 022431064) for further processing.

### Proteomics Sample Preparation and Nano LC-MS Analysis

The microdissected tissue samples were lysed and sonicated in lysis buffer containing 1% sodium dodecyl sulfate (SDS, Sigma-Aldrich), 50 mM triethylammonium bicarbonate (TEAB, Sigma-Aldrich), and 50 mM Tris-HCl at room temperature for 5 min. Cell lysates were reduced with freshly prepared 5 mM dithiothreitol (DTT, Thermo Fisher Scientific) at 56 °C for 30 min. Alkylation proteins were conducted by adding 20 mM iodoacetamide (IAA, Sigma-Aldrich) and incubating at room temperature for 30 min in the dark. After the reaction, protein samples were subjected to the single-pot, solid-phase enhanced sample-preparation (SP3) digestion protocol according to a previous study with minor modifications.^30^ Briefly, Sera-Mag carboxylate-modified hydrophilic beads were combined in a 1:1 ratio with hydrophobic beads (Cytiva, USA). Protein samples were mixed with prepared beads at a ratio of 1:10 (w/w), and protein binding was initiated by adding 100% ethanol to a 50% final concentration. The mixture was then incubated in a mixer at 1,000 r min^–1^ for 2 h at 37 °C. Subsequently, the binding buffer was removed on a magnetic rack. After washing beads with 80% ethanol, beads were resuspended in 50 mM TEAB supplemented with sequencing grade trypsin (Pierce) at a 1:50 enzyme/protein (w/w) ratio at 37 °C for 17 h. The digested peptides were desalted by C18 ZipTip pipet tips (Thermo Fisher Scientific, USA) and lyophilized in a freeze-dryer. The desalted peptides were resuspended in 0.1% fatty acid (FA) for the NanoLC-MS analysis.

LC-MS/MS analysis was conducted by using a timsTOF Pro mass spectrometer (Bruker Daltonics) connected to a nanoElute UPLC system (Bruker Daltonics) with a captive spray ion source. Peptides were loaded and separated on a 75 μm i.d. × 25 cm capillary column packed with C18 beads (1.6 μm, Aurora Series, IonOpticks) at a flow rate of 300 nL min^–1^, and the column temperature was maintained at 50 °C in a column oven. For all samples, the peptides were separated using a linear gradient from 2% to 17% phase B over 46.5 min, increased to 25% in 12 min, further increased to 37% in 7.5 min, and eventually raised to 95% in 9 min, followed by a 15 min wash at 95% phase B. Phase A consisted of 0.1% formic acid and 2% ACN in MS-grade water, while phase B comprised 0.1% formic acid in ACN. The sample was then analyzed under ddaPASEF mode with default parameters.^31^

### Data Analysis

MS raw data were searched using MaxQuant (Version 2.4.2.0, Max Planck Institute of Biochemistry, Germany) against the UniProt mouse database (2023, 17212 reviewed entries) with default setting for label-free quantitation (LFQ). The main search parameters used were as follows: Oxidation (M) and Acetyl (N-term) were set as variable modifications, and carbamidomethyl (C) was set as fixed modifications. Trypsin (P) was digestion enzyme. The minimal peptide length was set to 7, and the maximum miss cleavage was set to 2. Both protein identifications and peptide spectrum matches were subjected to a false discovery rate threshold of 1%. A Spearman’s correlation coefficient was calculated for all individual samples including the control group and PFOS exposure group. Proteins with 50% fold change compared to control (Fc > 1.5, or <0.67) and p-value <0.05 were defined as the significantly changed proteins. The student’s unpaired two-tailed *t* test calculated the P-value between the two groups. KEGG and GO enrichment analysis was performed using The Gene Ontology (GO) knowledgebase (http://geneontology.org) and String version 12.0 (https://string-db.org/) databases.^32^

### Metabolites and Lipids Extraction

Metabolite and lipid extraction from the microdissected tissue samples were performed as previously reported.^33^ The microdissected tissue samples were ultrasonically disrupted by a homogenizer (Xiaomei, XM-650DT) in 80 μL of ice-cold methanol and 20 μL of water. Subsequently, 60 μL of chloroform was added, and the homogenate was vortexed for 5 min. Then, 20 μL of water was added to promote phase separation. After equilibrating for 10 min, the mixture was centrifuged at 12,000 rpm for 15 min at 4 °C. The up and bottom layers were collected and transferred to new 0.5 mL tubes and dried with an IR concentrator (N-BIOTEK, NB-504CIR), respectively. Total protein content was quantified by the BCA assay (Thermo Fisher Scientific) for normalization. The Dried residuals of up and bottom were resuspended in 50 μL of methanol/water (1:1 v/v) and ACN/IPA/water (65:30:5 v/v/v) and then centrifuged at 8000g for 5 min at 4 °C before LC-MS/MS analysis, respectively. Quality control (QC) samples were constructed by mixing an equal amount (15 μL) of each sample and using this to monitor the whole sample set.

### Targeted Metabolomic Analysis

Targeted metabolomic analysis was performed as previously described.^34^ It was carried out on an Ultimate 3000 UHPLC system coupled with a triple-quadrupole (TSQ) mass spectrometer (Thermo Fisher Scientific). Metabolite separation was used with an ACQUITY UPLC BEH Amide column (2.1 × 100 mm, 1.7 μm, Waters) at a 0.3 mL min^–1^ flow rate and 30 °C column temperature. The mobile phase consisted of solvent A (10 mM ammonium acetate, 0.1% FA, in water) and solvent B (10 mM ammonium acetate, 0.1% FA, in ACN). The gradient was set as follows: 0 min, 100% B; 7 min, 70% B; 9.5 min, 40% B; 12 min, 30% B; 15 min, 100% B; 20 min, 100% B. Ten μL of sample was injected. The heated electrospray ionization source operated at a capillary voltage of −2.6 kV in negative ion mode. Sheath, aux, and sweep gas flow were set as 40, 10, and 1, respectively. The temperatures of capillary and probe were set as 350 and 320 °C. The precursor ion isolation window was set at ±0.35 *m*/*z* unit. The collision-induced dissociation (CID) gas was 1.5 mTorr with a well time of 50 ms. The identification of selected metabolites was based on the precise *m*/*z* and retention times of chemical standards. The summary of the detailed information for the detected metabolites and their MS/MS information is shown in Table S3. Data were processed by Thermo Xcalibur (version 4.1, Thermo Fisher Scientific). Relative metabolite levels were expressed relative to the levels of the control group after normalization to protein content.

## Results

### The Spatial Distribution of PFOS in Cardiac Sections

**Figure 1:**
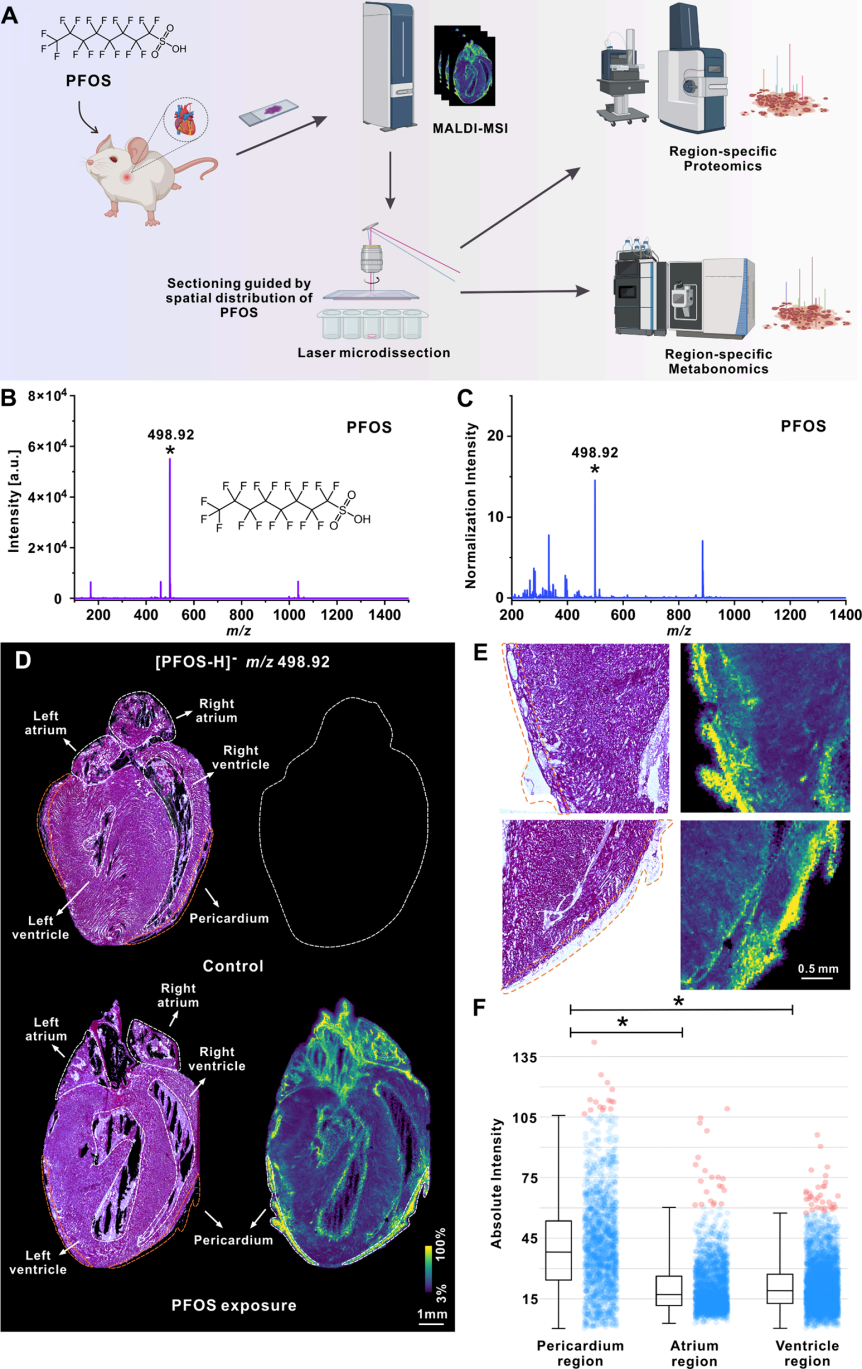
MALDI-MSI revealed spatial preference of PFOS accumulation in the mouse cardiac tissue. (A) The overall workflow of the study. (B) MALDI mass spectra of the PFOS standard ([M–H]^−^, *m*/*z* 498.92) were spotted on the steel plate under negative-ion detection mode. (C) Representative MALDI mass spectra of the PFOS ([M–H]^−^, *m*/*z* 498.92) spiking on the cardiac section under negative-ion detection mode. (D) Representative MALDI-MSI Image of PFOS distribution in cardiac sections from control and exposure groups. The optical micrograph of a H&E-stained parallel tissue section. Relative ion intensity is represented using a color bar. Orange dashed lines marked pericardium regions. (E) Representative pictures of enlarged pericardium areas (orange dashed lines) from adjacent slices upon PFOS exposure with H&E staining (left) and MALDI MSI (right). (F) Statistical analysis of PFOS peak intensities in different cardiac regions. (*) AUC > 0.75. Red dots represent outliers. Peak intensities were normalized to the total ion count.

Mouse cardiac tissue samples (n = 12, 6 mice each for the control and treatment group) from a previous PFOS exposure model were used.^25^ Mice in the exposure group were exposed to a 2 mg mL^–1^ concentration of PFOS for 14 days. MALDI-MSI was subsequently applied to analyze the spatial distribution of PFOS in cardiac tissue. Norharmane (Nor) was used as the MALDI matrix to detect PFOS in negative ionization mode.^28^ As shown in Figure 1B and Figure S2A, the characteristic ion peak of PFOS [M-H]^−^ in negative mode at *m*/*z* 498.92 could be detected with a relatively clean matrix background on the steel plate. The overview mass spectrum obtained after mass spectrometry imaging demonstrated that the PFOS peak could be distinctly observed in the tissue sections (Figure 1C). Additionally, there were no observable peaks in the range from *m*/*z* 450 to *m*/*z* 550, indicating no endogenous peaks that could interfere with the peak of PFOS in heart tissue (Figure S2C,D). Subsequently, the mass spectrometry imaging results of cardiac tissues after PFOS exposure are shown in Figure 1D. Of note, significant spatial distribution preference of PFOS was observed after acute exposure for 14 days, with no signal detected in the control group. Using the H&E-stained adjacent section as a reference, we found that PFOS signals were primarily concentrated in the pericardium, followed by the ventricular and atrial regions. In comparison, no PFOS signal was detected in the pericardial region of the control group (Figure 1D,E and Figure S3). A detailed examination of MSI data suggested that the PFOS distribution in the ventricular and atrial regions was mainly located in the ventricular and atrial walls. Quantitative analysis of normalized peak intensities showed that the PFOS signal in the pericardium was significantly higher than that in others (Figure 1F), indicating a preference for PFOS accumulation within a relatively short exposure period. Our data also unexpectedly revealed that the myocardial region seemed to be the less favorable region for PFOS accumulation, at least for the relatively short exposure period.

### Spatial Distribution Changes of Lipids in Cardiac Sections after PFOS Exposure Using MALDI-MSI

**Figure 2:**
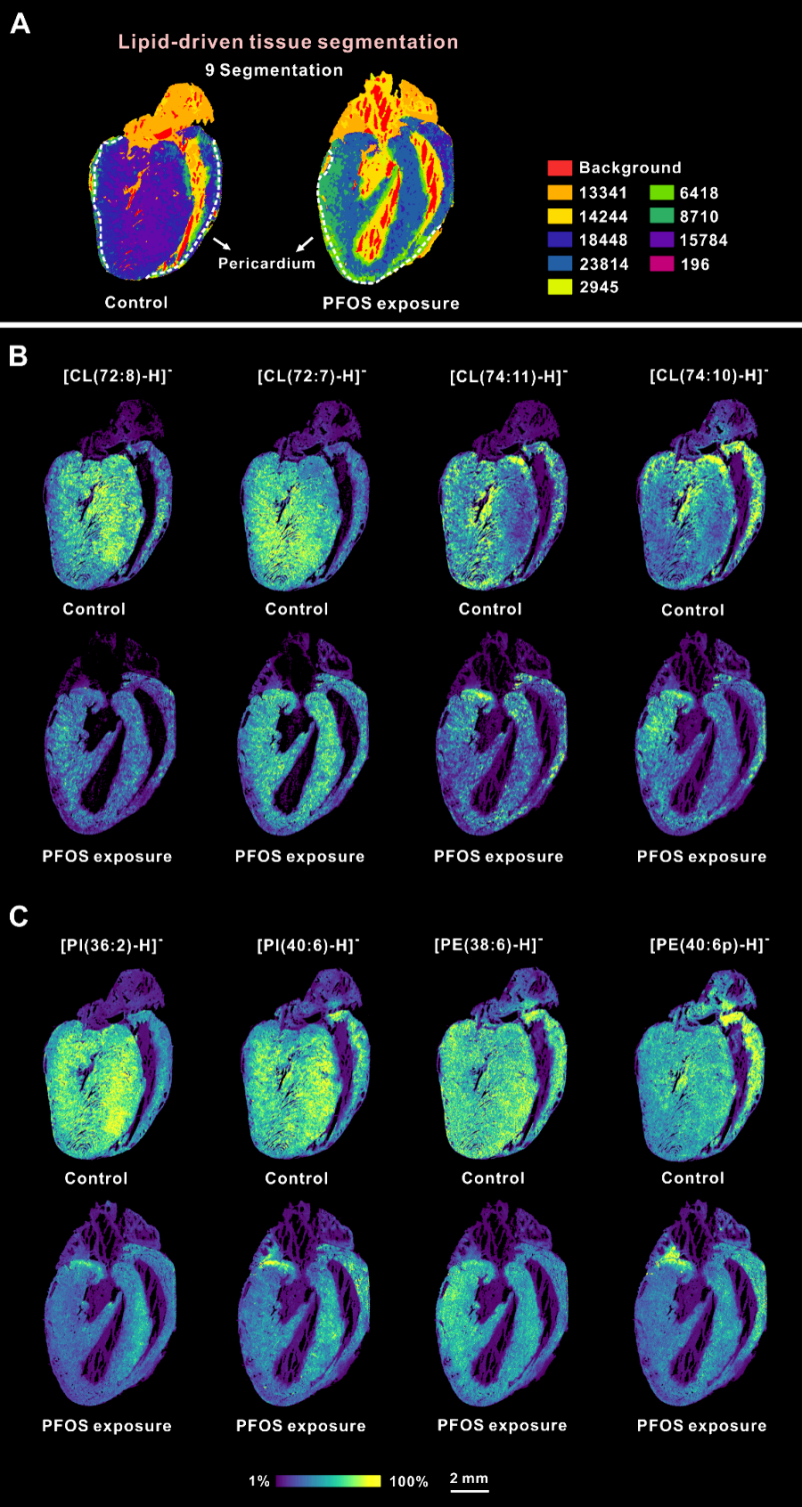
Representative MALDI-MSI images of altered lipids post PFOS exposure. (A) Segmentation map of cardiac sections based on the MALDI-MSI. The representative pericardium region was marked using white dashed lines. (B) Representative spatial distribution of the four cardiolipins in the cardiac sections. (C) Representative spatial distribution of phosphatidylinositol and phosphatidylethanolamine in the cardiac sections. Relative ion intensity is shown using a color bar. The intensity values were normalized to the TIC. All scale bars were 2 mm.

Using MALDI-MSI, the spatial-differential perturbation on the lipids of cardiac tissue upon PFOS exposure was visualized. We used NEDC as the matrix to enhance lipid detection sensitivity. A total of 46 lipid species were assigned and imaged in negative mode. The identified lipid species mainly included fatty acids (FAs), lysophosphatidylserines (LPSs), phosphatidylethanolamines (PEs), phosphatidylinositols (PIs), phosphatidylglycerols (PGs), and cardiolipins (CLs). The spatial distribution of lipids was found to be specific to the different cardiac anatomical regions. For example, cardiolipin was significantly higher in the myocardial tissue of the ventricles, and LPS seemed primarily distributed in association with blood. Bisecting k-means clustering was applied for unsupervised spatial segmentation to identify molecular species that differentiate between various tissue features. Tissue areas with similar lipid characteristics were clustered and assigned specific colors. As illustrated in Figure 2A, PFOS exposure induced apparent lipid composition change in the ventricular and the pericardium, which agreed with previous reports that PFOS exposure results in abnormal lipid metabolism.^19^ Notably, the pericardium region could be readily distinguished in both the control and exposure groups, suggesting a unique structural and molecular composition.

At the molecular level, we observed a significant decrease in the signal intensities of polyunsaturated phosphatidylinositol (PI) [PI(36:2)-H]^−^ and [PI(40:6)-H]^−^, as well as polyunsaturated long-chain phosphatidylethanolamine (PE) [PE(38:6)-H]^−^ and [PE(40:6p)-H]^−^, throughout the ventricular region (Figure 2C). In addition, Cardiolipin (CL) was found to be significantly affected by PFOS exposure. PFOS has been reported to decrease CL levels in hepatic cells; similarly, we observed this effect in the heart.^35^ Known for their crucial role in maintaining mitochondrial membrane structure, alterations in CL content are closely associated with numerous cardiovascular diseases. Upon PFOS exposure, the [CL(72:8)-H]^−^, [CL(72:7)-H]^−^, [CL(74:11)-H]^−^, and [CL(74:10)-H]^−^ were reduced in the ventricular region, with a more pronounced decrease near the pericardium (Figure 2B). As PFOS was primarily distributed in the pericardium where the CLs were reduced, a potential association of PFOS exposure-induced CL damage was observed.^35^ It is speculated that PFOS may insert into the mitochondrial outer membrane due to its high affinity to phospholipids and cause the effects.^36,37^

### Regional Resolution of Proteomics and Metabolomics Characterization in Cardiac Tissue upon PFOS Exposure

**Figure 3:**
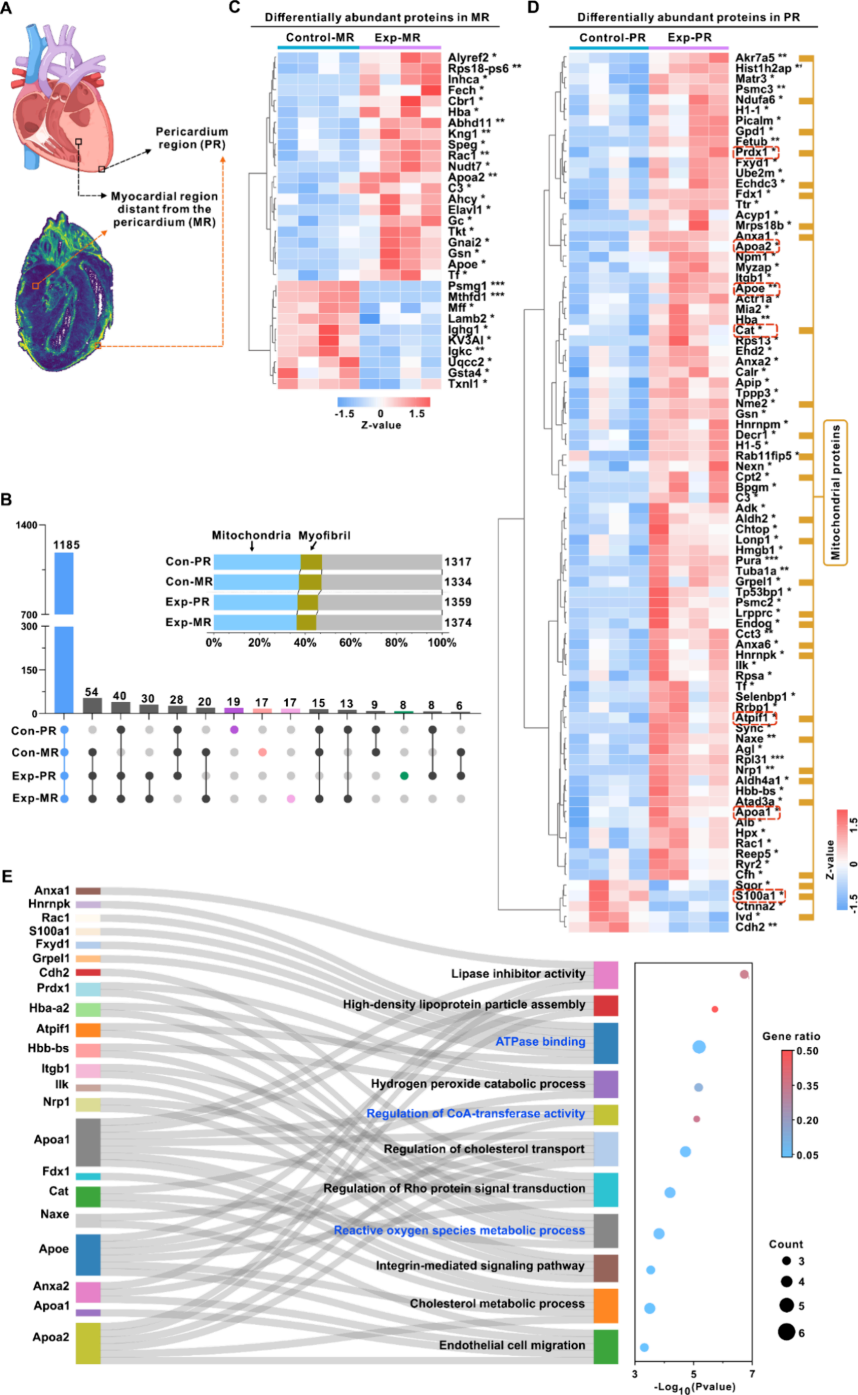
Region-specific dysregulation of protein expression in cardiac tissue upon PFOS exposure. (A) Schematic diagram of the selected regions of analysis by LMD. (B) UpSet plots illustrated the overlap of identified protein groups in each region. (C, D) Heatmap of the DEPs across MR region (C) and PR region (D) between the control and exposure group. The heatmap is based on the normalized abundance intensities of the significant DEPs by unsupervised hierarchical clustering. Statistical analysis used a two-tailed unpaired *t*-test; *p*-value is indicated for levels of significance *p-*value <0.01 (**), *p*-value <0.001 (***). (E) Sankey diagram of significantly enriched GO terms of the common DEPs of PR region between control and exposure samples. The sizes of dot indicate protein counts, and the colors represent the adjusted *p*-value. Abbreviations: PR, pericardium region; MR, myocardial region distant from the pericardium; Con-PR, the pericardium of the control group; Con-MR, the myocardium of the control group; Exp-PR, the pericardium of the PFOS exposure group; Exp-MR, the myocardium of the PFOS exposure group.

To further explore the region-specific differences in proteome levels caused by PFOS exposure, we isolated the pericardium region (PR) and myocardial region (MR) on the adjacent section slides via LMD (Figure 3A). The tryptic-digested peptides were then analyzed on a TimsTOF flex (Bruker) MS machine under the DDA mode. Label-free quantitation was performed by Maxquant (Version 2.4.2.0) against the mouse Uniprot database. A total of 1317 and 1334 proteins were identified in the PR and MR areas of the control group, while 1359 and 1374 proteins were identified in the corresponding regions of the exposure group, respectively. Upset plots showed that the proteins uniquely identified in the PR of the control group, MR of the control group, MR of the exposed group, and PR of the exposed group were 19, 17, 17, and 8, respectively (Figure 3B). Cellular component analysis revealed that the identified proteins were primarily associated with mitochondria and myofibrils (Figure 3B). We identified eight proteins exclusive to the exposure group PR, with leucine-rich single-pass membrane protein 2 (LSMEM2) and MRPL2 showing significant associations with cardiac diseases. LSMEM2 is a cell surface N-glycoprotein predominantly expressed in cardiomyocytes, implicated in cell–cell adhesion and the chemomechanical coupling of these cells. Recent studies have highlighted its association with heart failure.^38^ Another important protein is MRPL2, a key member of the mitochondrial ribosomal family of proteins, which plays a pivotal role in mitochondrial protein synthesis, mitochondrial metabolic regulation, ATP production, and mitochondrial DNA transcription. Dysregulation of MRPL2 expression has been linked to the pathogenesis of cardiac diseases.^39^

Differentially expressed proteins (DEPs) were then selected based on the following criteria: a 50% change in fold change (FC) (>1.5 or <0.67) and p-value <0.05 for a two-tail Student’s *t* test. The results showed that the number of DEPs in the PR (79 upregulated, 5 downregulated) was significantly higher than in the MR (21 upregulated, 10 downregulated) post-PFOS exposure (Figure 3C,D), indicating that PR is the primary site of action for PFOS exposure. Gene Ontology (GO) enrichment analysis was then conducted on these DEPs. No pathway was enriched in the MR. In contrast, a set of pathways was found to be enriched in PR, including regulation of cholesterol transport, ATPase binding, regulation of CoA-transferase activity, and the reactive oxygen species metabolic process (Figure 3E).

Consistent with previous studies, our findings indicate that PFOS affects lipid transport and cholesterol homeostasis in PFOS-accumulated PR. We identified three key members of the apolipoprotein family: ApoE (FC = 5.52, p = 0.001), ApoA1 (FC = 1.83, p = 0.049), and ApoA2 (FC = 2.35, p = 0.018) were upregulated in the PR of the PFOS exposure group. These apolipoproteins are major components of high-density lipoprotein (HDL) and very-low-density lipoprotein (VLDL). Their abnormal upregulation may increase the risk of cardiovascular diseases such as atherosclerosis and pericarditis.^40,41^ Moreover, we found significant expression changes in two key proteins involved in mitochondrial function: S100A1 (FC = 0.04, p = 0.036) and ATPIF1 (FC = 2.74, p = 0.015). S100A1 is a calcium-binding protein that regulates mitochondrial energy metabolism, mitochondrial fusion and fission processes, and apoptosis by controlling calcium ion concentration within mitochondria.^42^ A reduction in S100A1 can lead to mitochondrial energy metabolism imbalance, increased ROS production, and impaired cell function.^43^ ATPIF1 primarily maintains cellular energy balance and mitochondrial function by regulating ATP synthase activity.^44^ The upregulation of ATPIF1 serves as a mitochondrial protective mechanism under oxidative stress, inhibiting ATP hydrolysis activity of ATP synthase, thereby adjusting energy metabolism, maintaining mitochondrial membrane potential, and reducing ROS production.^45^ Additionally, we observed significant upregulation of Prdx1 (FC = 1.77, p = 0.032) and Cat (FC = 1.57, p = 0.033), two crucial enzymes in maintaining intracellular and mitochondrial redox balance. Mitochondrial dysfunction can lead to excessive ROS production, and the upregulation of Prdx1 and Cat may act as protective mechanisms by reducing oxidative stress, thus preserving mitochondrial biomolecules and associated metabolic pathways from oxidative damage.^46,47^ In conclusion, our proteomics data reveal that the spatial distribution and accumulation of PFOS profoundly influence protein expression in cardiac tissues. Specifically, in the PFOS-accumulated PR, proteins affected by PFOS are implicated in lipid transport, cholesterol homeostasis, and mitochondrial dysfunction.^19,48^ These observed results are consistent with previous transcriptomic studies on rat cardiac tissue following PFOS exposure, which reported that PFOS damages heart mitochondria and limits mitochondrial ATP production.^26^ It is worth noting that in the myocardial region where the PFOS concentration was markedly lower no significant changes in related protein expression were detected.

**Figure 4:**
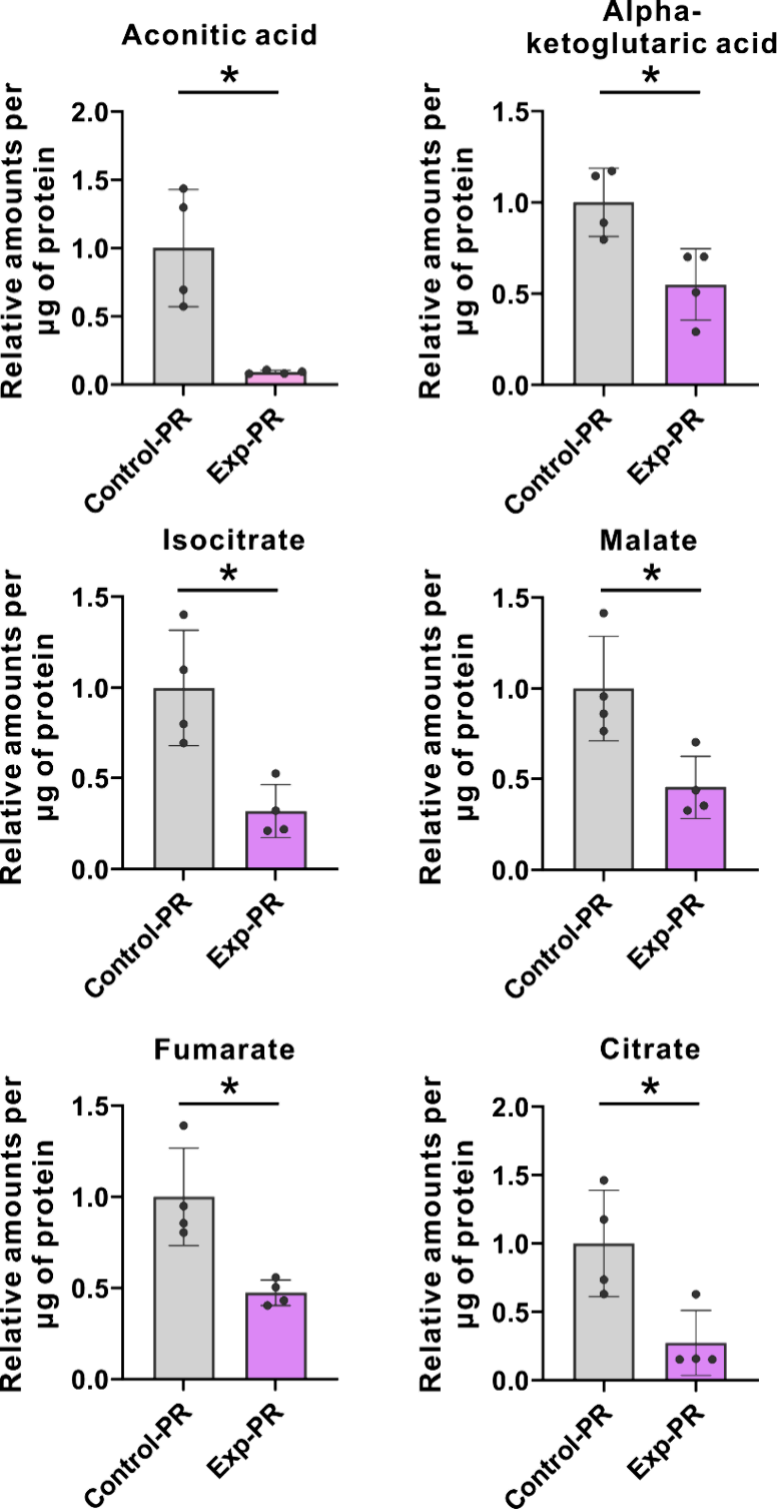
Impairment of TCA cycle activity in PR region following PFOS exposure. Targeted relative quantitation of metabolites in the PR region is associated with glycolysis and TCA cycle, with/without the exposure of PFOS. Statistical significance was calculated using the unpaired two-tailed Student’s *t* test. *P < 0.05; **P < 0.01.

Following this, we further validated the mitochondrial dysfunction induced by PFOS in the PR region by targeted metabolomics analysis, regarding key energy metabolic pathways. The results indicated that PFOS exposure led to a significant downregulation of 10 metabolites in the PR samples, including citrate, aconitic acid, isocitrate, alpha-ketoglutaric acid, fumarate, and malate, which are all key intermediates of the mitochondrial tricarboxylic acid (TCA) cycle (Figure 4 and Figure S4). These findings suggest that PFOS exposure impairs the TCA cycle activity in the PR region. However, in the MR region samples, the differentially abundant metabolites resulting from PFOS exposure were not primarily associated with the TCA cycle, with only alpha-ketoglutaric acid, an intermediate of the TCA cycle, being downregulated (Figure S5). This is consistent with our proteomics data, which indicate that accumulation of PFOS in the PR region could induce mitochondrial dysfunction, thereby reducing TCA cycle efficiency. In summary, both proteomic and metabolomic showed a spatial differential response upon PFOS exposure and correlated with the observed accumulation pattern of PFOS in cardiac tissues.

## Discussion

Extensive prior research has elucidated the biodistribution and toxicity of PFOS in various biological systems; however, investigations at the suborgan level remain scarce. In this study, we present the first to employ spatial multiomics to reveal the distribution and corresponding mechanism of PFOS in cardiac tissue. Our data reveal that PFOS exhibits a heterogeneous spatial distribution within cardiac tissues, predominantly accumulating in the pericardium, resulting in differential toxicological impacts across cardiac substructures.

The pericardium consists of two layers: the sturdy fibrous pericardium, providing structural support, and the inner serous pericardium, which includes a parietal layer attached to the fibrous pericardium and a visceral layer adhering to the epicardium.^49^ These layers encase the pericardial cavity, containing lubricating pericardial fluid.^50^ We hypothesized that two major factors contribute to PFOS accumulation in the pericardium. First, the pericardial fluid is mainly from plasma ultrafiltration and contains a high concentration of albumin (approximately 70%),^50^ the primary binding molecule of PFOS in the animal. The pericardial fluid therefore contains a relatively high PFOS level. The second reason is that PFOS is easily absorbed and integrated into the phospholipid bilayers of cell membranes, becoming trapped within cells. Consequently, PFOS carried by albumin in the pericardial fluid may partition into the cell membrane lipids within the pericardial region, leading to its accumulation there. Again, PFOS’s dual affinity properties to albumin and phospholipid contributed to its accumulation in the pericardium.

**Figure 5:**
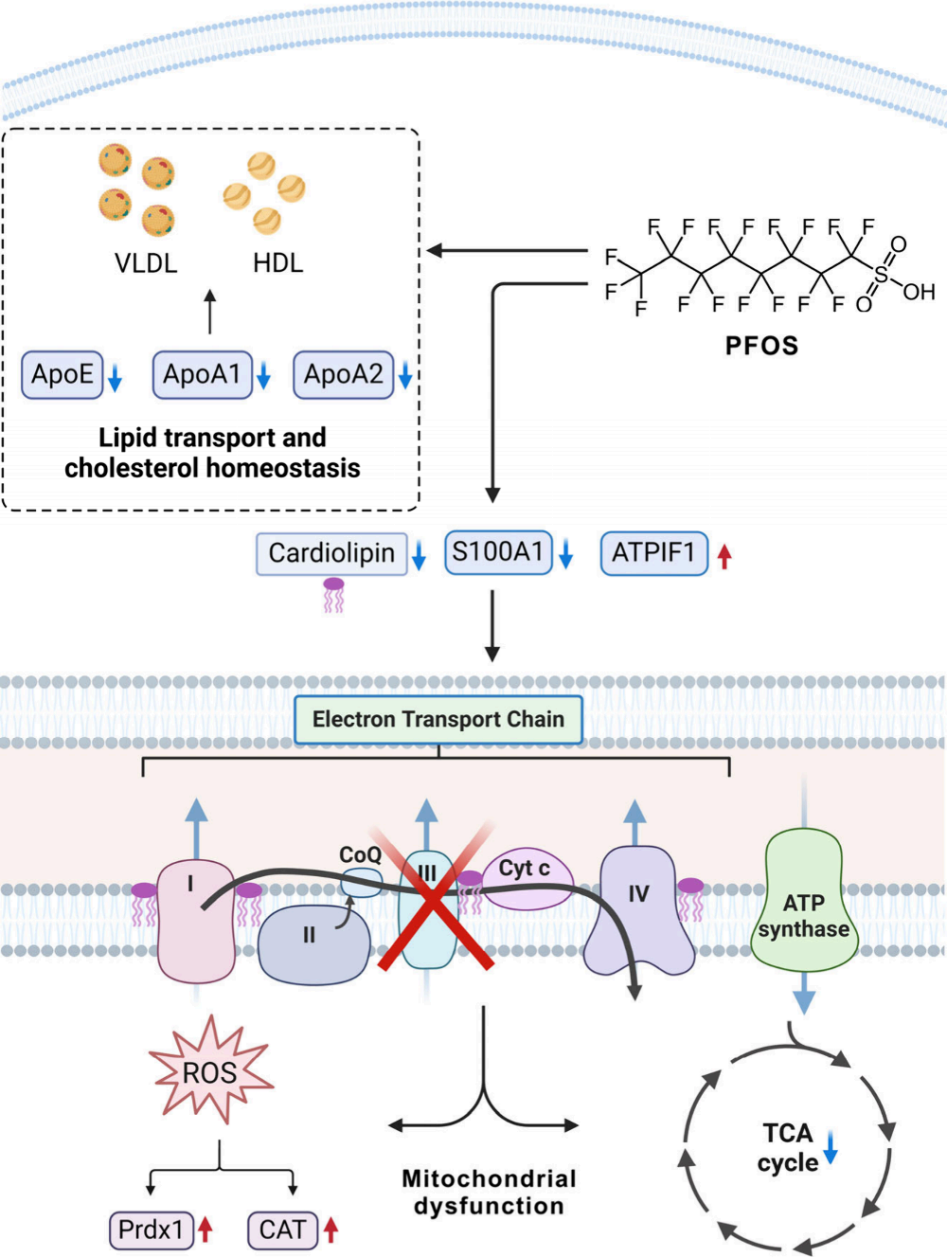
Schematic diagram to illustrate the mechanisms that impact lipid transport and cholesterol homeostasis and induce mitochondrial dysfunction.

Recent studies have highlighted the significant tissue heterogeneity in the heart, revealing notable differences in cell types, tissue microenvironments, and molecular levels across various anatomical regions.^51,52^ This heterogeneity thus might contribute to the differential distribution of environmental pollutants as well as region-specific responses to environmental pollutant molecules. However, conventional tissue homogenization coupling with LC-MS analysis would lose this information. In this study, we developed an MS-based spatial multiomics workflow to address this challenge. The integration of MALDI-MSI technology has brought significant advantages, as it provides spatial distribution information on environmental pollutants within tissues in a label-free manner. In this study, to observe bioaccumulation and subsequent toxic responses in a relatively short time, 2 mg/kg PFOS was applied. The concentration was based on previous PFOS toxicity studies,^25,26^ without causing death. A longer period of exposure with the same dose (19 days at 2 mg/kg) showed no significant change in body weight or obvious damage to heart tissue by H&E staining. We mapped the spatial distribution of PFOS in the heart and revealed a previously unknown preferential accumulation of PFOS in the pericardium rather than the myocardium (Figure 1D). This spatial distribution information on PFOS accurately guided our subsequent spatial multiomics investigations using LMD. By isolating the pericardium and myocardial regions from PFOS-exposed mouse hearts using LMD, we observed different molecular changes in the cells of these regions. PFOS buildup in the pericardium significantly disrupts the area’s lipid profile, particularly reducing cardiolipin—a vital mitochondrial lipid essential for electron transport chain (ETC) function and mitochondrial integrity. This reduction impairs the ETC, affecting oxidative phosphorylation and energy metabolism, akin to the effects seen in Barth syndrome (Figure 5). The proteomic analysis also reveals that PFOS alters protein expression in the pericardium, specifically upregulating enzymes such as Prdx1 and Cat that counteract oxidative stress, signaling mitochondrial distress. This finding is consistent with previous work reported by Xia,^26^ which showed an upregulation of uncoupling proteins (UCP1 and UCP3) that serve as protective mechanisms against PFOS-induced ROS. Xia’s research further showed that subunits of the ATP synthase complex, including ATP5E, ATP5I, and ATP5O, were downregulated, a process directly linked to mitochondrial damage and reduced cellular energy production. In parallel, our proteomic analysis identified a significant upregulation of ATPIF1, an inhibitor that suppresses both ATP synthase and hydrolase activities, thereby exacerbating the reduction in mitochondrial ATP synthesis.^53,54^ Mitochondrial dysfunction was further validated by targeted metabolomics, demonstrating a repression of the TCA cycle in the pericardium following PFOS exposure. The results from spatial lipidomics, LMD-based proteomics, and targeted metabolomics were in line with each other and collectively demonstrated that PFOS exposure induced mitochondrial dysfunction in the pericardium. These findings are of critical importance for the development of therapeutic strategies against PFOS-induced cardiac toxicity and tissue damage.

The MS-based functional spatial omics workflow that we proposed could be technically demanding and resource-intensive. It cannot analyze pollutants unsuitable for MALDI-MSI, such as ozone. Obtaining fine slides from hard tissues like bone poses challenges, although new sample pretreatment methods are emerging to address this issue.^55^ A significant limitation is the restricted spatial resolution, which hinders the detailed analysis of fine tissue structures at the single-cell level. Currently, commercially available instruments provide a resolution limit of 5 to 10 μm,^56,57^ while achieving matrix crystallite sizes below 1 μm remains difficult.^58^ However, the advent of expansion microscopy offers promising opportunities to enhance imaging resolution and subsequent proteomics analysis.^57,59,60^ Further research is needed to effectively incorporate this technique while balancing finer spatial resolution with an adequate signal intensity.

In conclusion, MS-based spatial omics could serve as a powerful tool for uncovering the distribution and bioaccumulation of environmental pollutants. It also enables us to investigate the molecular mechanism of pollutants with spatial resolution, leading to novel discoveries. Furthermore, our spatial multiomics workflow ensures systematic data collection while exhibiting significant flexibility and versatility, offering valuable insights for future spatial omics research on various environmental pollutants.

## Supporting Information Available

Figure S1: Histological images of H&E staining sections of the pericardium and myocardium regions of the heart were collected from the control and PFOS-treated groups. Figure S2: Mass spectra of Norharmane (Nor) obtained on the steel plate or heart section in negative ion mode. Figure S3: Representative pictures of enlarged pericardium areas on adjacent slices from the control group with H&E staining and MALDI MSI. Figure S4: The differentially expressed metabolites in the PR region are associated with glycolysis and TCA cycle. Figure S5: The differentially expressed metabolites in the MR region are associated with glycolysis and TCA cycle. Figure S6: The correlation matrix for normalized protein abundances across the individual samples. Table S1: Assigned differentially expressed lipids in both positive and negative modes. Table S2: The specific proteins associated with Exp-PR. Table S3: The list of detailed information and MRM transitions for the metabolites. Table S4: The list of abbreviations (PDF)
