# Supplementary material for: Mass Spectrometry-Based Spatial Multiomics Revealed Bioaccumulation Preference and Region-Specific Responses of PFOS in Mice Cardiac Tissue
Source: Environ Sci Technol. 2025 Jan 22;59(4):1957–68. doi: 10.1021/acs.est.4c09874 (PMC11800377; doi:10.1021/acs.est.4c09874)
Supplement: Supplementary file 1 — es4c09874_si_001.pdf [file es4c09874_si_001.pdf]

Supporting Information

Mass Spectrometry-based Spatial Multi-omics Revealed Bioaccumulation Preference and Region-specific Responses of PFOS in Mice Cardiac Tissue

Rui Shi[1†], Yanyan Chen[1†], Wenlong Wu[1†], Xin Diao[1], Leijian Chen[1], Xingxing Liu[1], Haijiang Wu[1], Jianing Wang[1], Lin Zhu[1*], Zongwei Cai[1,2*]

[1]State Key Laboratory of Environmental and Biological Analysis, Hong Kong Baptist University, Hong Kong SAR 999077, China.

[2]Eastern Institute of Technology, Ningbo 315200, China

†These authors contributed equally to this work.

*Corresponding author.

Corresponding author:

Lin Zhu - Email: zhu_lin@hkbu.edu.hk

Zongwei Cai - Email: zwcai@hkbu.edu.hk

This Supporting Information includes:

14 pages, 6 figures, 4 tables.

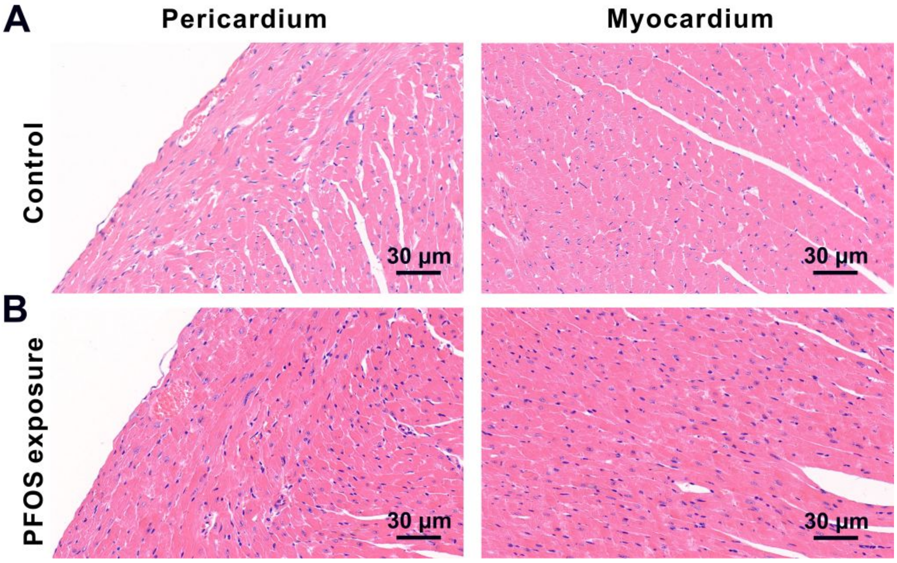

Figure S1. Histological images of H&E staining sections of the pericardium and myocardium regions of the heart were collected from the control (A) and PFOS-treated groups (B).

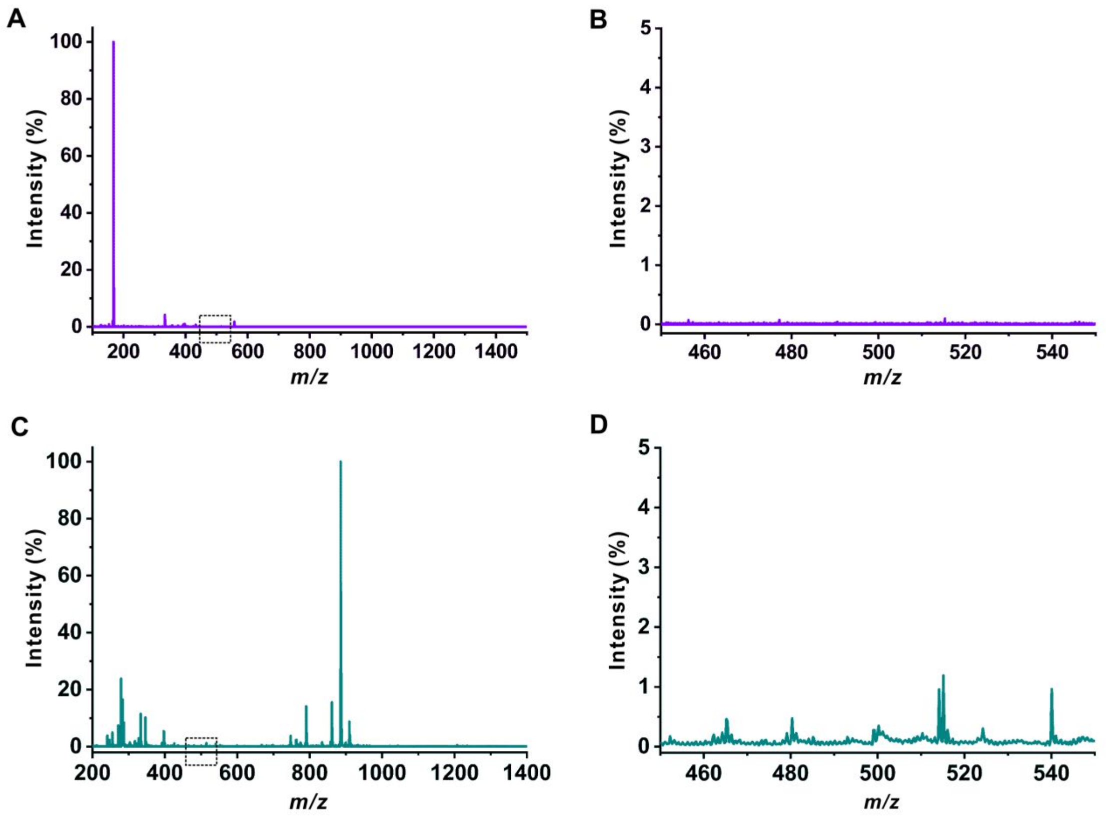

Figure S2. Mass spectra of Norharmane (Nor) obtained on the steel plate or heart section in negative ion mode. (A) MALDI mass spectra of the Nor without analyte under negative-ion detection mode on the steel plate. (B) Zoomed-in MALDI mass spectra for the range of m/z 450 to m/z 550 in Panel (A). (C) MALDI mass spectra were directly obtained from the heart section without PFOS under negative-ion detection mode. (D) Zoomed-in MALDI mass spectra for the range of m/z 450 to m/z 550 in Panel (C). The peak of the highest intensity was set as 100%.

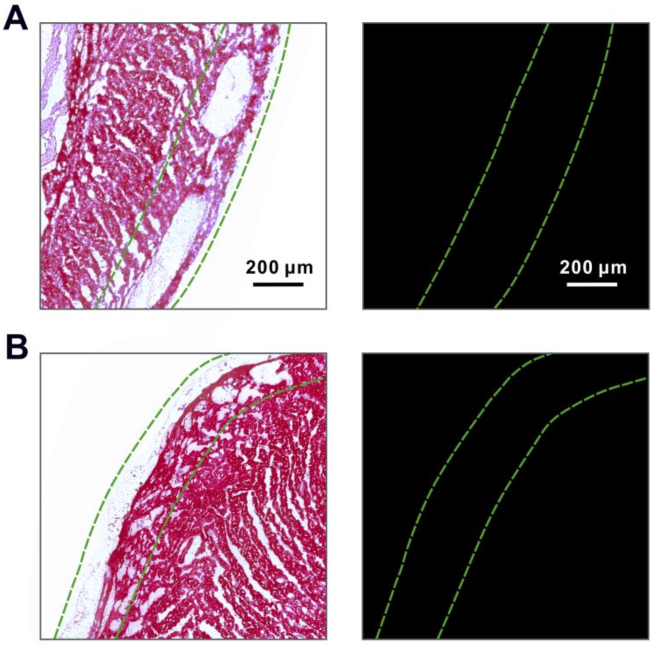

Figure S3. Representative pictures of enlarged pericardium areas (green dashed lines) on adjacent slices from the control group with H&E staining (left) and MALDI MSI (right).

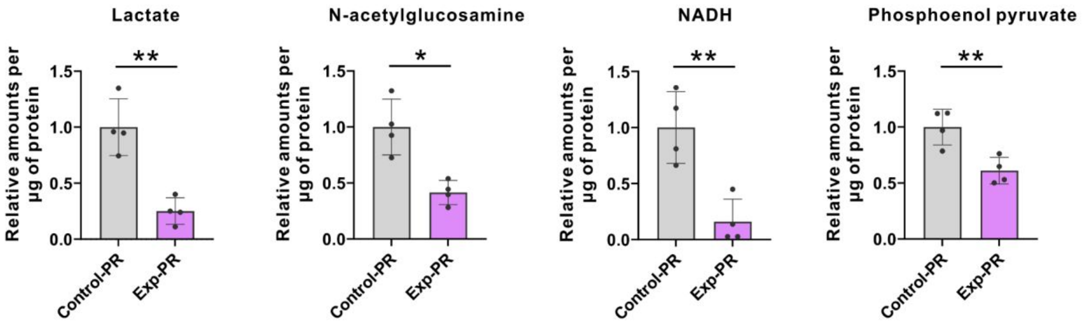

Figure S4. The differentially expressed metabolites in the PR region are associated with glycolysis and the TCA cycle, with/without exposure to PFOS. Statistical significance was calculated using the unpaired two-tailed Student's t-test. *P < 0.05; **P < 0.01.

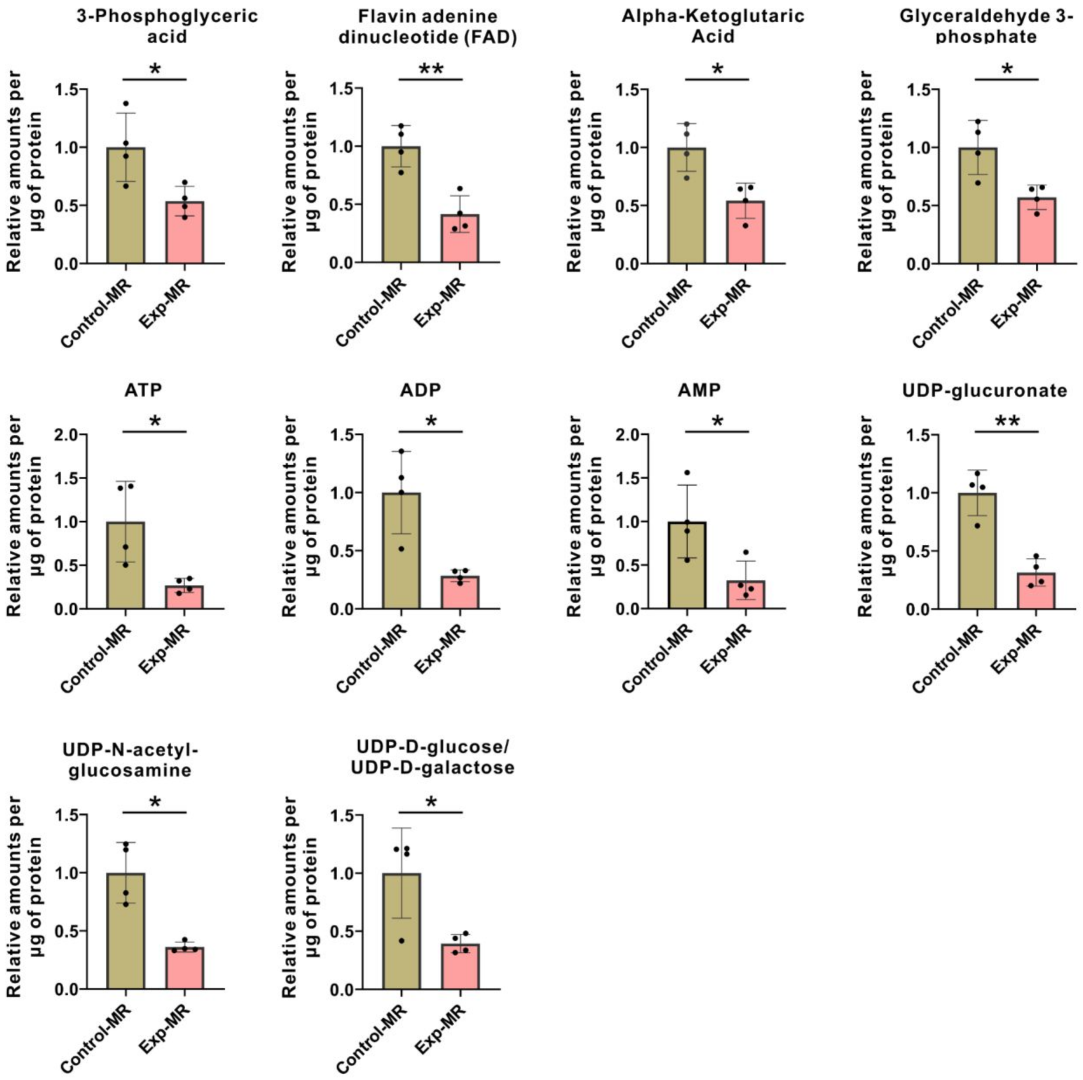

Figure S5. The differentially expressed metabolites in the MR region are associated with glycolysis and the TCA cycle, with/without exposure to PFOS. Statistical significance was calculated using the unpaired two-tailed Student's t-test. *P < 0.05; **P < 0.01.

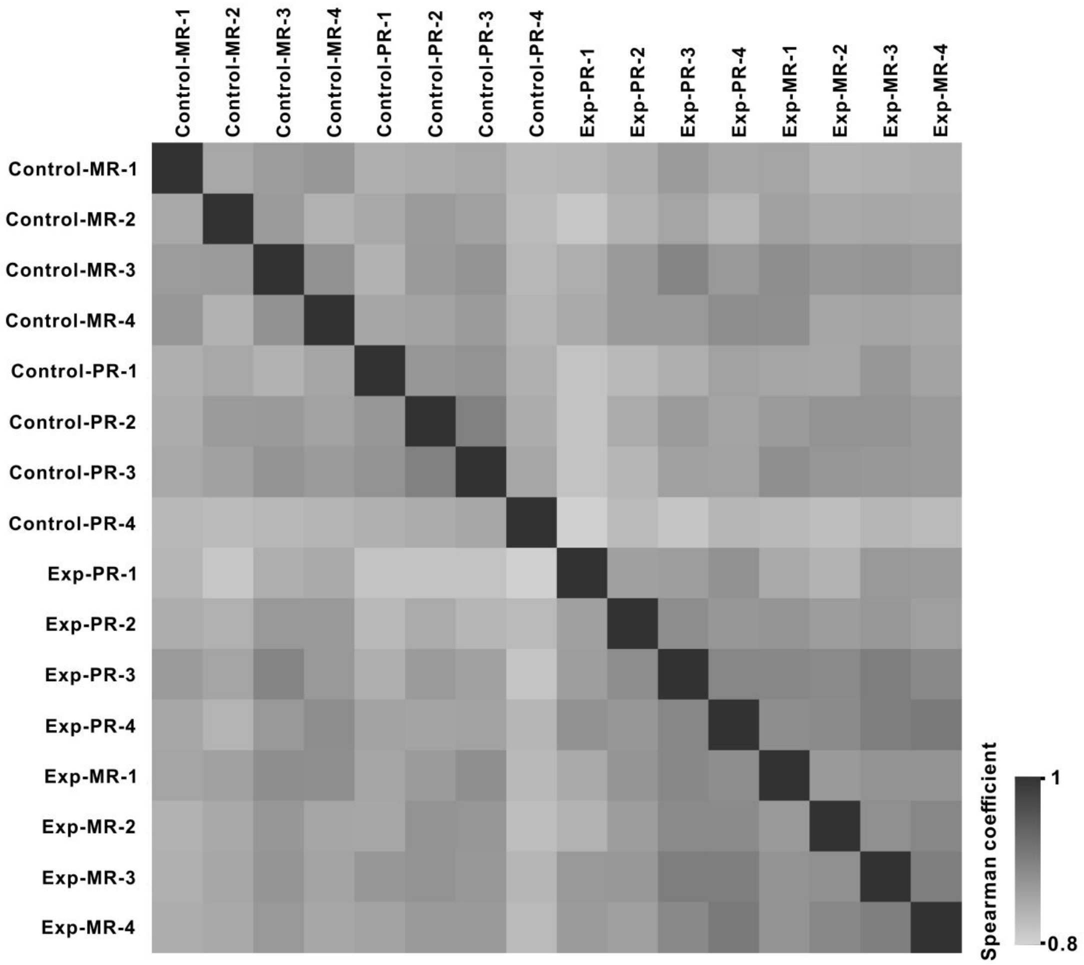

Figure S6. Graphical representation of the correlation matrix for normalized protein abundances across the individual samples: the Spearman rank correlation coefficient is color-coded. Using a p-value < 0.0001 as a cutoff for significance.

Table S1 Assigned lipids in negative ion mode.

Assignment	Ion type	Experimental m/z	Theoretical m/z	Mass error (ppm)
FA 16:4; O2	$[M-H]^-$	279.158	279.1602	7.88
FA 18:1	$[M-H]^-$	281.247	281.2486	5.69
FA 18:0	$[M-H]^-$	283.263	283.2632	0.71
FA 20:4	$[M-H]^-$	303.234	303.233	3.30
FA 22:6	$[M-H]^-$	327.231	327.233	6.11
FA 33:1	$[M-H]^-$	491.479	491.4834	8.95
LPA 22:0	$[M-H]^-$	493.333	493.33	6.08
FA 32:0; O	$[M-H]^-$	495.475	495.4783	6.66
LPS 18:0	$[M-H]^-$	524.296	524.2994	6.48
LPS 20:2	$[M-H]^-$	548.296	548.2994	6.20
LPC 20:0	$[M-H]^-$	550.393	550.3878	9.45
LPS 20:0	$[M-H]^-$	552.328	552.3307	4.89
LPC 21:4	$[M-H]^-$	556.346	556.3409	9.17
PI 18:0/OH	$[M-H]^-$	599.315	599.3197	3.72
PE 16:0/18:1	$[M-H]^-$	716.52	716.5228	7.84
PE 16:0p/20:4	$[M-H]^-$	722.51	722.5125	3.91
PE 18:1p/18:1	$[M-H]^-$	726.54	726.5438	3.46
PE 18:0/18:1	$[M-H]^-$	744.548	744.5543	5.23
PE 16:0p/22:6	$[M-H]^-$	746.51	746.5119	8.46
PG 16:0/18:1	$[M-H]^-$	747.51	747.5161	2.55
PE 38:6	$[M-H]^-$	762.501	762.5079	8.16
PE 18:0/20:4	$[M-H]^-$	766.532	766.5387	9.05
PE 18:0p/22:6	$[M-H]^-$	774.536	774.5432	8.74
PS 18:0/18:1	$[M-H]^-$	788.548	788.5442	9.30
PE 18:0/22:6	$[M-H]^-$	790.531	790.5381	4.82
PS 18:0/22:6	$[M-H]^-$	834.524	834.5286	8.98
PI 36:4	$[M-H]^-$	857.515	857.5186	5.51
PI 36:2	$[M-H]^-$	861.544	861.5499	4.20
PI 18:1/20:4	$[M-H]^-$	883.533	883.5337	6.85
PI 38:4	$[M-H]^-$	885.543	885.5499	0.79
PI 38:3	$[M-H]^-$	887.5688	887.5655	7.79
PI 40:6	$[M-H]^-$	909.558	909.5499	8.91
CL 72:8	$[M-H]^-$	1447.963	1447.965	1.38
CL 72:7	$[M-H]^-$	1449.992	1449.981	7.59
CL 72:6	$[M-H]^-$	1452.004	1451.996	5.51

CL 74:11	$[M-H]^-$	1469.937	1469.949	8.16
CL 74:10	$[M-H]^-$	1471.977	1471.967	6.79
CL 74:9	$[M-H]^-$	1473.999	1473.985	9.50
CL 74:8	$[M-H]^-$	1475.996	1475.999	2.03
CL 76:12	$[M-H]^-$	1495.984	1495.97	9.36
CL 76:11	$[M-H]^-$	1497.989	1497.981	5.34
CL 76:10	$[M-H]^-$	1500.006	1499.996	6.67
CL 78:15	$[M-H]^-$	1517.939	1517.949	6.59
CL 78:14	$[M-H]^-$	1519.954	1519.965	7.24
CL 78:13	$[M-H]^-$	1521.973	1521.981	5.26
CL 78:12	$[M-H]^-$	1523.986	1523.996	6.56

63 **Table S2 The specific proteins associated with Exp-PR.**

**Entry Name**	**Protein names**	**Gene names**
A0A571BE98_MOUSE	Leucine-rich single-pass membrane protein 2	Lsmem2
ACTN4_MOUSE	Alpha-actinin-4 (Non-muscle alpha-actinin 4)	Actn4
IASPP_MOUSE	RelA-associated inhibitor (Inhibitor of ASPP protein) (Protein iASPP) (NFkB-interacting protein 1)	Ppp1r13l Nkip1
H2B1C_MOUSE	Histone H2B type 1-C/E/G	H2bc4 Hist1h2bc; H2bc6 Hist1h2be; H2bc8 Hist1h2bg
WDR37_MOUSE	WD repeat-containing protein 37	Wdr37 Kiaa0982
NUDC3_MOUSE	NudC domain-containing protein 3	Nudcd3 Kiaa1068
CLUA1_MOUSE	Clusterin-associated protein 1	Cluap1
RM02_MOUSE	Large ribosomal subunit protein uL2m (39S ribosomal protein L2, mitochondrial) (L2mt) (MRPL2)	Mrpl2

Table S3 The list of detailed information and MRM transitions for the metabolites.

Metabolites	HMDB ID	Detection mode	R.T. (min)	Precursor Ion (*m/z*)	Product ion[1] (*m/z*)	Product ion[2] (*m/z*)	MS/MS information (*m/z*)
2-Hydroxyglutarate	HMDB0000694	Negative	9.86	147	129	57	130, 129, 109, 101, 103, 86, 85, 80, 58, 57, 55
3-Phosphoglyceric acid	HMDB0000807	Negative	10.42	185	97	79	167, 97, 79, 68
Aconitic acid	HMDB0000072	Negative	10.23	173	85	129	129, 85, 84, 83, 67, 41
Adenosine triphosphate	HMDB0000538	Negative	10.52	506	408	159	478, 408, 383, 280, 192, 159, 156, 82, 72, 64, 58,
Adenosine 5'-diphosphate	HMDB0001341	Negative	10.36	426	159	328	408, 346, 328, 291, 273, 245, 214, 193, 159, 134, 120, 97, 79, 62
Alpha-ketoglutaric acid	HMDB0000208	Negative	9.49	145	57	101	134, 126, 113, 107, 101, 87, 83, 73, 57
Adenosine monophosphate	HMDB0000045	Negative	10.16	346	79	134	299, 211, 193, 151, 134, 107, 97, 89, 79, 74
Citric acid	HMDB0000094	Negative	10.63	191	87	111	173, 155, 147, 129, 111, 103, 88, 87, 85, 76, 67, 57
Flavin adenine dinucleotide	HMDB0001248	Negative	9.79	784	97	437	517, 455, 437, 408, 346, 273, 255, 241, 211, 195, 181, 177,163, 159,130, 97, 79
Fructose-1,6-bisphosphate	HMDB0001058	Negative	10.71	339	241	97	241, 169, 151, 139, 97, 79
Fructose-6-phosphate	HMDB0000124	Negative	10.34	259	241	79	241, 223, 199, 169, 139, 125, 101, 97, 79, 73
Fumaric acid	HMDB0000134	Negative	9.61	115	71	45	114 104 72 71 62 59 53 45

Glucose	HMDB0003345	Negative	8.54	179	89	71	179, 172, 161, 151, 141, 119, 113, 112, 101, 95, 89, 85, 83, 73, 71
Glucose-1-phosphate	HMDB0001586	Negative	10.36	259	169	79	169, 139, 125, 101, 97, 87, 79, 70
Glucose-6-phosphate	HMDB0001401	Negative	10.48	259	199	79	241, 199, 161, 139, 127, 101, 97, 79, 68
Glyceraldehyde 3-phosphate	HMDB0001112	Negative	10.16	169	97	79	151, 99, 97, 79
Isocitric acid	HMDB0000193	Negative	10.41	191	111	117	173, 155, 129, 117, 111, 99, 85, 73, 67, 57, 41
Lactate	HMDB0144295	Negative	7.94	89	43	41	78, 75, 69, 59, 43, 41
Malate	HMDB0000156	Negative	9.93	133	115	71	116, 115, 104, 94, 89, 81, 74, 73, 71, 68
N-acetyl-glucosamine	HMDB0000215	Negative	9.01	220	59	97	142, 119, 97, 89, 83, 73, 71, 59, 58
N-Acetylglucosamine phosphate	HMDB0002817	Negative	10.27	300	79	97	282, 264, 252, 234, 199, 184, 166, 154, 142, 97, 79
NADH	HMDB0001487	Negative	9.87	664	408	79	428, 408, 346, 328, 273, 262, 211, 159, 144, 93, 91, 79
NADP+	HMDB0000217	Negative	10.54	742	620	408	620, 602, 506, 426, 408, 291, 273, 254, 239, 184, 159, 137
Nicotinamide adenine dinucleotide	HMDB0000902	Negative	10.24	662	540	328	540, 447, 426, 408, 359, 346, 328, 273, 247, 211, 193, 175
Oxaloacetate	HMDB0000223	Negative	9.68	131	87	59	130, 87, 86, 59, 46, 44, 42
Phosphoenol pyruvate	HMDB0000263	Negative	10.38	167	79	139	139, 125, 118, 100, 97, 90, 86, 82, 79, 78, 69, 63

Pyruvate	HMDB0000243	Negative	6.25	87	43	87	87, 43
Succinate	HMDB0000254	Negative	9.74	117	73	99	99, 73, 72, 59, 55
UDP-D-galactose	HMDB0000302	Negative	10.31	565	323	79	565, 547, 403, 385, 323, 321, 305, 280, 273, 241, 211, 97
UDP-glucuronate	HMDB0000935	Negative	10.49	579	403	79	534, 403, 316, 255, 159, 111, 92, 88, 79, 56
UDP-N-acetyl-glucosamine	HMDB0000290	Negative	10.18	606	385	79	447, 403, 385, 362, 323, 300, 282, 273, 261, 209, 177, 175,159, 111, 109, 97, 92, 79, 61

Table S4. The list of abbreviations

Abbreviations	Full name
PFAS	per-and polyfluoroalkyl substances
PFOS	perfluorooctane sulfonate
MALDI-MSI	Matrix-assisted laser desorption/ionization-mass spectrometry imaging
LMD	laser microdissection
LC/MS	liquid chromatography-mass spectrometry
H&E	Hematoxylin and Eosin
Nor	Norharmane
NEDC	*N*-naphthylethylenediamine dihydrochloride
FA	fatty acids
LPS	lysophosphatidylserines
PE	phosphatidylethanolamines
PI	phosphatidylinositols
PG	phosphatidylglycerols
CL	cardiolipins
PR	pericardium region
MR	myocardial region distant from the pericardium
DEPs	Differentially expressed proteins
LFQ	label-free quantitation
LSMEM2	leucine-rich single-pass membrane protein 2
HDL	high-density lipoprotein
VLDL	very low-density lipoprotein
TCA cycle	tricarboxylic acid cycle
ETC	electron transport chain
NADH	reduced nicotinamide adenine dinucleotide
NADP+	nicotinamide adenine dinucleotide phosphate
ATP	adenosine triphosphate
ROS	reactive oxygen species
TIC	total ion count
GO	Gene Ontology
KEGG	Kyoto Encyclopedia of Genes and Genomes
ACN	acetonitrile
SDS	sodium dodecyl sulfate
IAA	iodoacetamide
